# Hypoxia promotes histone H3K9 lactylation to enhance *LAMC2* transcription in esophageal squamous cell carcinoma

**DOI:** 10.1016/j.isci.2024.110188

**Published:** 2024-06-05

**Authors:** Yong Zang, Aiyuan Wang, Jianji Zhang, Mingxin Xia, Zixin Jiang, Bona Jia, Congcong Lu, Chen Chen, Siyu Wang, Yingao Zhang, Chen Wang, Xinyi Cao, Ziping Niu, Chaoran He, Xue Bai, Shanshan Tian, Guijin Zhai, Hailong Cao, Yupeng Chen, Kai Zhang

**Affiliations:** 1 The Province and Ministry Co-sponsored Collaborative Innovation Center for Medical Epigenetics, Key Laboratory of Immune Microenvironment and Disease (Ministry of Education), Tianjin Key Laboratory of Medical Epigenetics, Department of Biochemistry and Molecular Biology, School of Basic Medical Sciences, Tianjin Medical University, Tianjin 300070, China; 2 Frontier Center for Cell Response, College of Life Sciences, Nankai University, Tianjin 300071, China; 3 Department of Gastroenterology and Hepatology, Tianjin Medical University General Hospital, Tianjin Institute of Digestive Diseases, Tianjin Key Laboratory of Digestive Diseases, Tianjin, China; 4 Tianjin Key Laboratory of Ionic-Molecular Function of Cardiovascular Disease, Department of Cardiology, Tianjin Institute of Cardiology, The Second Hospital of Tianjin Medical University, Tianjin, China; 5 Tianjin Institute of Urology, The Second Hospital of Tianjin Medical University, Tianjin Medical University, Tianjin, China; 6 Tianjin Key Laboratory of Retinal Functions and Diseases, Eye Institute and School of Optometry, Tianjin Medical University Eye Hospital, Tianjin Medical University, Tianjin 300070, China

**Keywords:** cancer, epigenetics, proteomics, transcriptomics

## Abstract

Hypoxia promotes tumorigenesis and lactate accumulation in esophageal squamous cell carcinoma (ESCC). Lactate can induce histone lysine lactylation (Kla, a recently identified histone marks) to regulate transcription. However, the functional consequence of histone Kla under hypoxia in ESCC remains to be explored. Here, we reveal that hypoxia facilitates histone H3K9la to enhance *LAMC2* transcription for proliferation of ESCC. We found that global level of Kla was elevated under hypoxia, and thus identified the landscape of histone Kla in ESCC by quantitative proteomics. Furthermore, we show a significant increase of H3K9la level induced by hypoxia. Next, MNase ChIP-seq and RNA-seq analysis suggest that H3K9la is enriched at the promoter of cell junction genes. Finally, we demonstrate that the histone H3K9la facilitates the expression of *LAMC2* for ESCC invasion by *in vivo* and *in vitro* experiments. Briefly, our study reveals a vital role of histone Kla triggered by hypoxia in cancer.

## Highlights

• The landscape of histone lactylation was identified in ESCC cells under hypoxia
• To identify the target genes of histone H3K9la induced by hypoxia in KYSE30 cells
• Hypoxia promotes histone H3K9la to enhance LAMC2 transcription for ESCC progression
• A hypoxia-induced, Kla-dependent mechanism for transcription and tumorigenesis

## Graphical abstract

**Figure:**
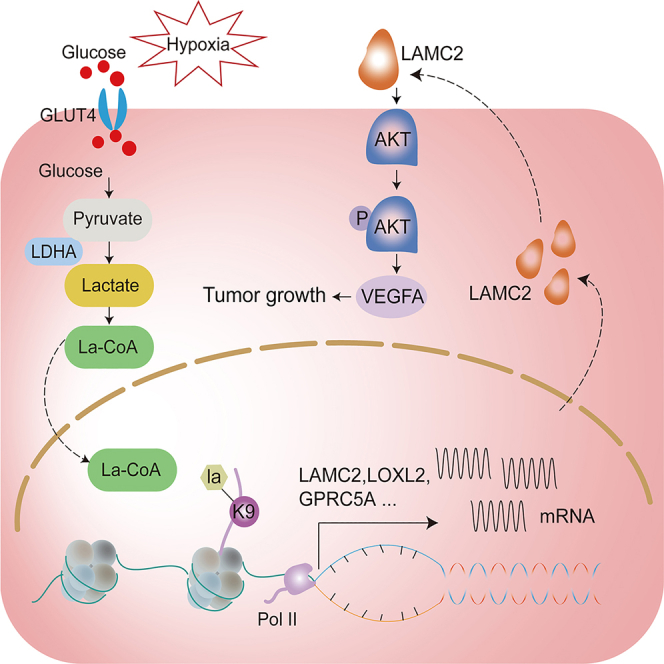

## Introduction

Hypoxia has been considered as one common characteristic of tumors, resulting in lactate production and accumulation in tumor microenvironment.^1^ As the end product of glucose metabolism, lactate has long been considered as a waste product from cellular respiratory metabolism under hypoxic conditions.^2^ However, growing evidence indicates that lactate is not only a major energy source and gluconeogenic precursor, but also an important signaling molecule, serving both metabolic and non-metabolic functions in the tumor microenvironment.^3^

The recently identified lysine lactylation (Kla) has provided a new insight into the mechanism of lactate regulation. Histone Kla, which is induced by lactate could directly regulate gene transcription and polarization of macrophage.^4^ Subsequent researches suggest that Kla is involved in the regulation of multiple biological processes, including immunosuppression, oncogenesis, and metabolic reprogramming.^5^^,^^6^^,^^7^ However, the role of histone Kla in cancers remains to be explored.

Esophageal carcinoma (EC) is one of the most aggressive cancers worldwide and responds poorly to therapy.^8^^,^^9^ In 2020, EC has caused 0.6 million new cases and 0.54 million deaths worldwide.^10^ As the major sub-type of this cancer (over 90%), esophageal squamous cell carcinoma (ESCC) is highly aggressive with a five-year survival rate of only 10%.^11^ The low survival rate is largely due to a lack of deep understanding in mechanisms of ESCC tumorigenesis. Accumulated studies have shown that hypoxia promotes ESCC progression and tumorigenesis by stimulating tumor glycolysis.^12^^,^^13^^,^^14^ Furthermore, hypoxia-induced genes is significantly related to the poor prognosis of ESCC patients.^15^^,^^16^ In order to survive in solid ESCC tumors, where is hypoxic microenvironments, ESCC cells can reprogram metabolic activities.^17^^,^^18^ Notably, glycolysis, which has been widely recognized as the primary method of tumor cells energy metabolism,^19^^,^^20^^,^^21^^,^^22^ not only provides energy for tumor cells, but also lactate.^23^^,^^24^ However, it is currently unclear what effect the accumulated lactate has on ESCC cells.

Here, we found that global level of Kla was obviously elevated in ESCC under hypoxia. Using Kla affinity enrichment and isotope-labeled quantitative proteomics, we explored the landscape of histone Kla and found a significant increase of H3K9la level induced by hypoxia. MNase ChIP-seq analysis showed that H3K9la was enriched at the promoter of cell junction genes. RNA-seq analysis also confirmed that the-related genes were increased under hypoxia. Finally, we identified that the H3K9la facilitates the expression of *LAMC2* and confirmed its contribution to ESCC migration and invasion through *in vitro* and *in vivo* experiments. Briefly, our study revealed a transcription regulation mediated by hypoxia-trigged histone H3K9 lactylation for tumorigenesis in ESCC.

## Results

### Hypoxia promotes ESCC progression and induces metabolic reprogramming

In tumor cells, lactate, which is enhanced due to the increased glycolytic rate and hypoxic conditions, has been recognized as not only a major energy source, but also as a key signaling molecule, serving non-metabolic functions.^25^ However, the function and regulatory mechanism of lactate are still not clear enough. The newly discovered histone lysine lactylation (Kla) provide a new insight into mechanism of lactate regulation. Here, we have performed the screening for an immortalized normal esophageal epithelium (NE3) and multiple esophageal cancer cell lines (KYSE30, KYSE140, KYSE150, and KYSE450), western blot showed that the histone Kla levels in KYSE30 cells were higher compared to other cell lines (Figure S1A). So, we chose the KYSE30 cells as a model for studying histone Kla on ESCC behaviors.

**Figure 1:**
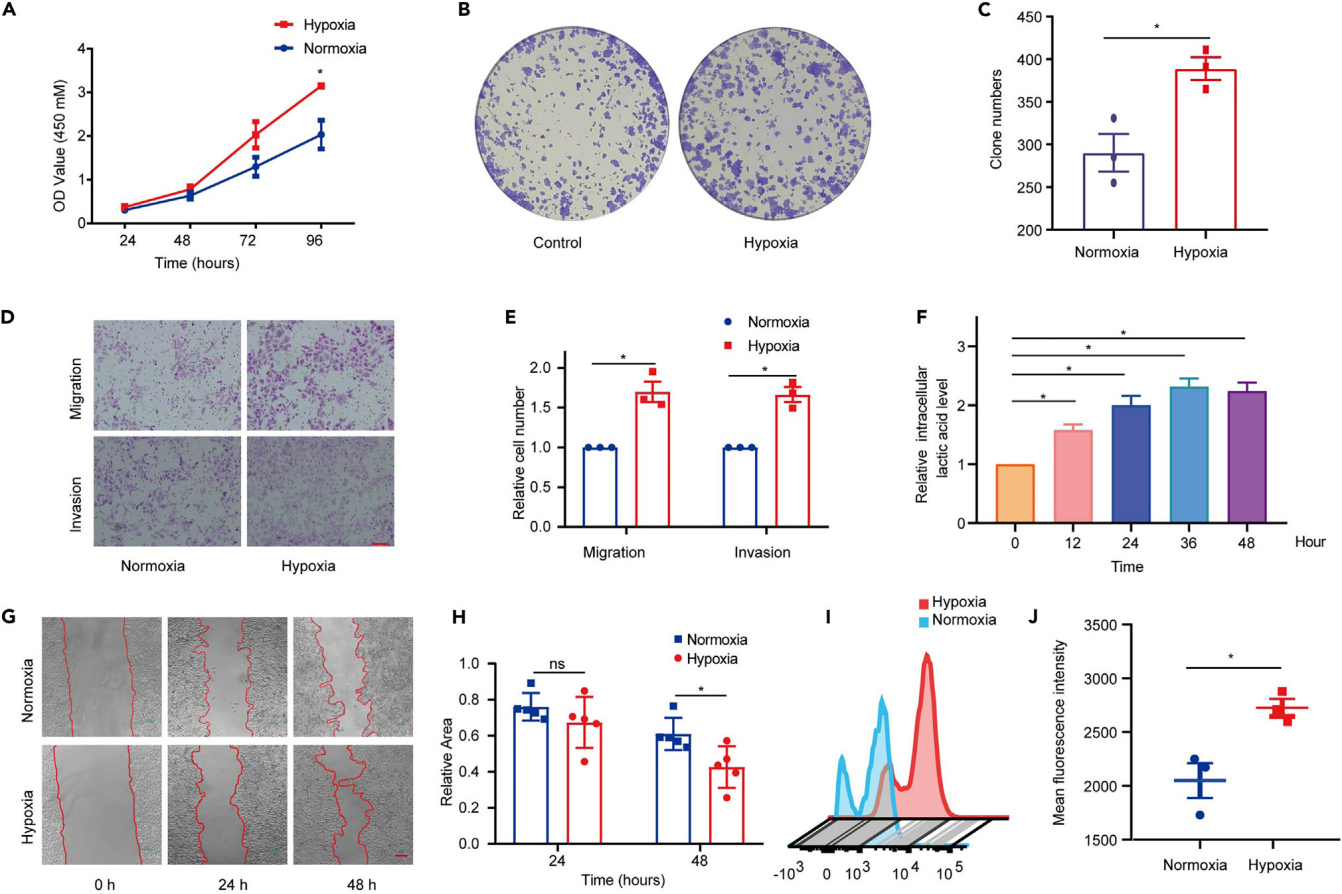
Hypoxia promoted ESCC cells progression and influenced metabolic reprogramming (A) CCK-8 assay showed that hypoxia significantly promoted the cell viability (*n* = 3 per group). (B and C) Hypoxia increased the colony formation rate in KYSE30 cells (*n* = 3 per group). (D and E) Hypoxia significantly elevated KYSE30 cells migration and invasion. The scale bar shows 100 μm (*n* = 3 per group). (F) The intracellular lactic acid level was increased when KYSE30 cells cultured in hypoxia environment (*n* = 3 per group). (G and H) Hypoxia promoted KYSE30 cells motility. The scale bar shows 100 μm (*n* = 5 per group). (I and J) Hypoxia significantly increased glucose uptake in KYSE30 cells (*n* = 3 per group). Statistical significance was analyzed by *Student’s t-test*. Data are mean ± SEM. ∗*p* < 0.05.

Hypoxia is one of the defining features of tumor microenvironment. To test the effect of hypoxia on ESCC progression, KYSE30 cells were cultured under normoxia (21% O_2_) and hyproxia (1% O_2_), respectively. CCK-8 assay showed that hypoxia obviously promoted the cell viability (Figure 1A). Meanwhile, we found that hypoxia induced an increase of colony formation rate of KYSE30 cells (Figures 1B and 1C). Furthermore, hypoxia obviously elevated KYSE30 cells migration and invasion (Figures 1D and 1E). Besides, wound healing assay showed that hypoxia promoted KYSE30 cells motility (Figures 1G and 1H).

Previous studies have shown that ESCC cells tend to utilize glycolysis to produce energy. In glycolysis, glucose is converted to lactate by metabolic enzymes rather than to acetyl-coenzyme A, which is the material of tricarboxylic acid (TCA) cycle.^26^ In light of the metabolic reprogramming changed in ECSS cells under hypoxia, we examined the levels of intracellular and secreted lactate and measured glucose uptake in differently cultured KYSE30 cells. And thus, we observed that the levels of intracellular and secreted lactate were both gradually increased under hypoxia compared to that under normoxia (Figure 1F and S1B). Glucose uptake was also increased in KYSE30 cells determined by 2-NBDG assay (Figures 1I, 1J, and S1C). Together, these results indicate that hypoxia induces the metabolic reprogramming in ESCC cells and promotes the malignant phenotype.

### The landscape of histone lactylation triggered by hypoxia in ESCC cells

**Figure 2:**
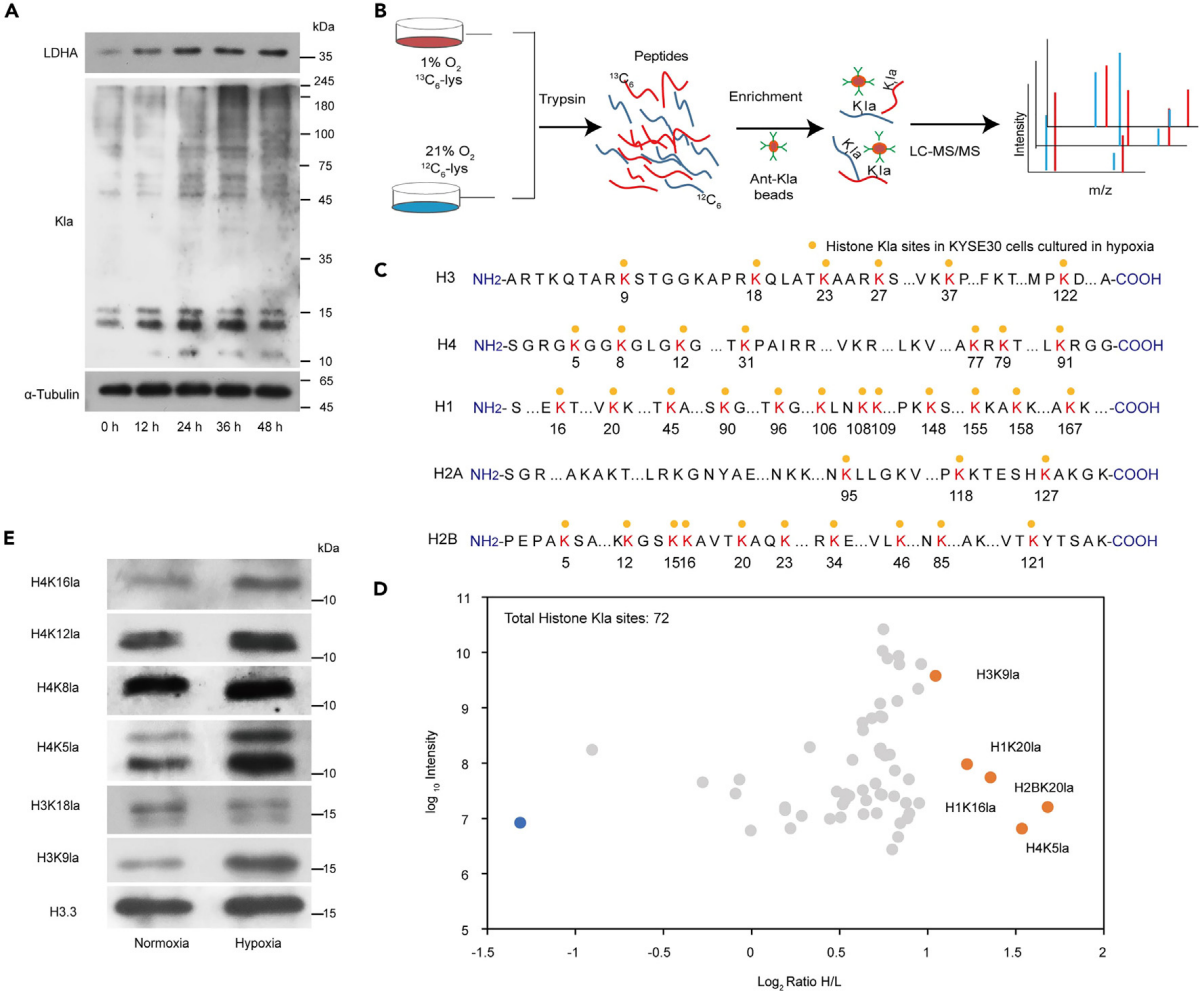
The landscape of histone lactylation triggered by hypoxia in ESCC cells (A) Global Kla level was increased in KYSE30 cells cultured in hypoxia environment. (B) The experimental workflow of SILAC quantitative proteomics to analyze the landscape of histone Kla regulated by hypoxia in the in KYSE30 cells. (C) Illustration of histone Kla sites identified in KYSE30 cells cultured in hypoxia environment. (D) Scatterplot showing the ratio of Kla proteins in KYSE30 cells cultured in hypoxia versus normoxia. (E) The Kla level of H4K16, H4K12, H4K8, H4K5, H3K18, and H3K9 in KYSE30 cells were increased in hypoxia.

Lactate, which is an important substrate for Kla, has increased under hypoxia. To explore the relationship between hypoxia and Kla in KYSE30 cells. Here, we performed western blotting to analyze the Kla level in KYSE30 cells. We identified that the global Kla level was gradually increased in KYSE30 cells under hypoxia culture (Figure 2A). Recent evidence suggests that histone Kla plays an important role in gene expression regulation.^5^^,^^27^ To identify potential histone Kla sites trigged by hypoxia in ESCC, we performed a stable isotope labeling by amino acids in cell culture (SILAC)-based quantitative proteomic analysis coupled with Kla affinity enrichment to characterize the landscape of histone Kla in the KYSE30 cells (Figures 2B, 2C, S2A, and S2B). A total of 72 Kla sites were identified on histone proteins (Figure 2D and Table S1). By comparing the KYSE30 cells cultured in normoxia, we show that hypoxia induces a general increase of histone Kla level, where 5 Kla sites are most significant (more than a 2×increase), including H2BK21, H4K5, H1K16, H1K20, and H3K9 (Figure 2D). Accumulated studies have shown that post-translational modifications (PTMs) on H3K9, such as methylation and acetylation have very important functions in regulation of gene transcription,^28^^,^^29^ and thus, we focused on H3K9la. After validating the increase of H3K9la in hypoxia (Figures 2E and S2C), we reasonably assumed that H3K9la is a potential marker triggered by hypoxia in KYSE30 cells.

### Genome-wide analysis of the transcriptional consequences of H3K9la in KYSE30 cells cultured under hypoxia

**Figure 3:**
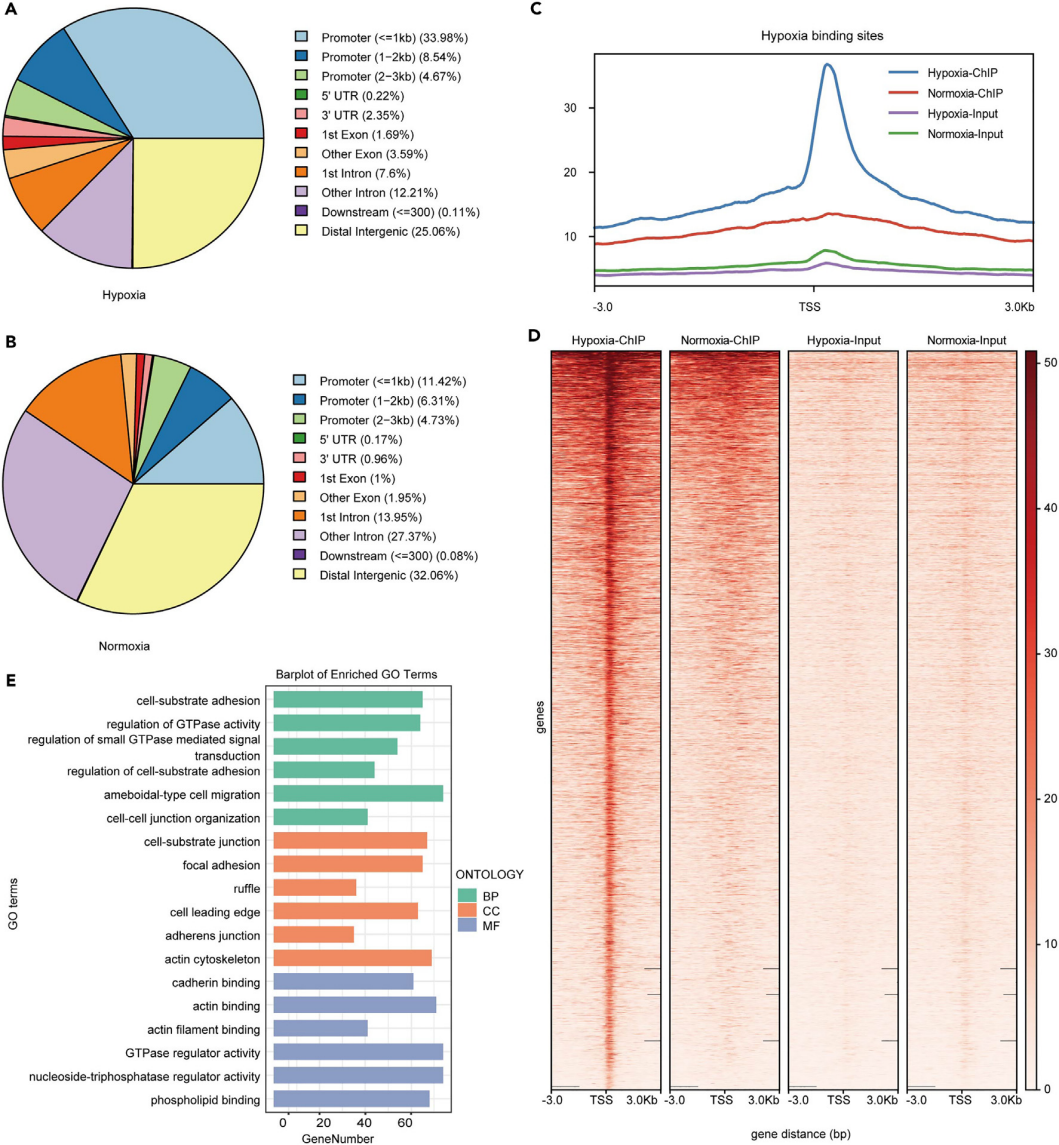
Genome-wide analysis of the transcriptional consequences of H3K9la in KYSE30 cells cultured in normoxia and hypoxia (A and B) Genome-wide distribution of H3K9la-binding peaks in KYSE30 cells cultured in hypoxia versus normoxia or normoxia versus hypoxia. (C and D) The peaks and heatmaps presents the signal intensity, which were enrich under hypoxia on the different H3K9la binding peaks in KYSE30 cells cultured hypoxia and normoxia, ordered by signal strength. ChIP-seq enrichment profiles around central position of H3K9la binding sites. (E) GO enrichment analysis of the elevated H3K9la binding different peaks at candidate target genes.

Histone PTMs are crucial regulators of chromatin structure and gene expression. To further investigate the function of H3K9la in ESCC under hypoxia, we performed MNase ChIP-seq analysis to explore candidate genes modulated by H3K9la in KYSE30 cells cultured in 1% O_2_ or 21% O_2_, resulting that a total of 3624 and 2408 peaks were found in the control and hypoxia group, respectively (Figures 3A and 3B). The informatics analysis further revealed that enrichment of peak was near the transcription start sites (TSS) (Figures 3C and 3D). Notably, H3K9la was enriched at the promoters (<3Kb) of enriched genes with 47.19% peaks in hypoxia group and 22.1% peaks in normoxia group (Figures 3A and 3B).

To further determine the potential function of H3K9la in ESCC cells in hypoxia. We carried out gene ontology (GO) enrichment analysis and Kyoto Encyclopedia of Genes and Genomes (KEGG) pathway enrichment analysis for H3K9la binding genes (of 2224 genes), which were enriched in hypoxia group. As shown in Figure 3E, cell-substrate adhesion, regulation of GTPase activity and regulation of small GTPase-mediated signal are listed in the top 3 GO terms in biological process (BP); cell-substrate junction, focal adhesion, and ruffle are listed in the top 3 GO terms in cellular component (CC); cadherin binding, actin binding, and actin filament binding were in the top 3 GO terms in molecular function (MF). Meanwhile, the results of KEGG pathway enrichment analysis showed that H3K9la binding genes were mostly enriched in “phosphatidylinositol-3-kinase (PI3K)-Akt signaling pathway”, “human papillomavirus infection”, “mitogen activated protein kinase (MAPK) signaling pathway”, “focal adhesion” and “regulation of actin cytoskeleton” (Figure S3A). Notably, the called peaks identified candidate genomic loci cell-junction genes, such as *GPRC5A*, *LOXL2*, *EFEMP2*, and *ITGA5* (Figures S3B–S3E). From previous analysis, we noticed that H3K9la binding genes in hypoxia were associated with cell junction and cell structure.

### RNA-seq analysis combined with H3K9la MNase ChIP-seq in KYSE30

**Figure 4:**
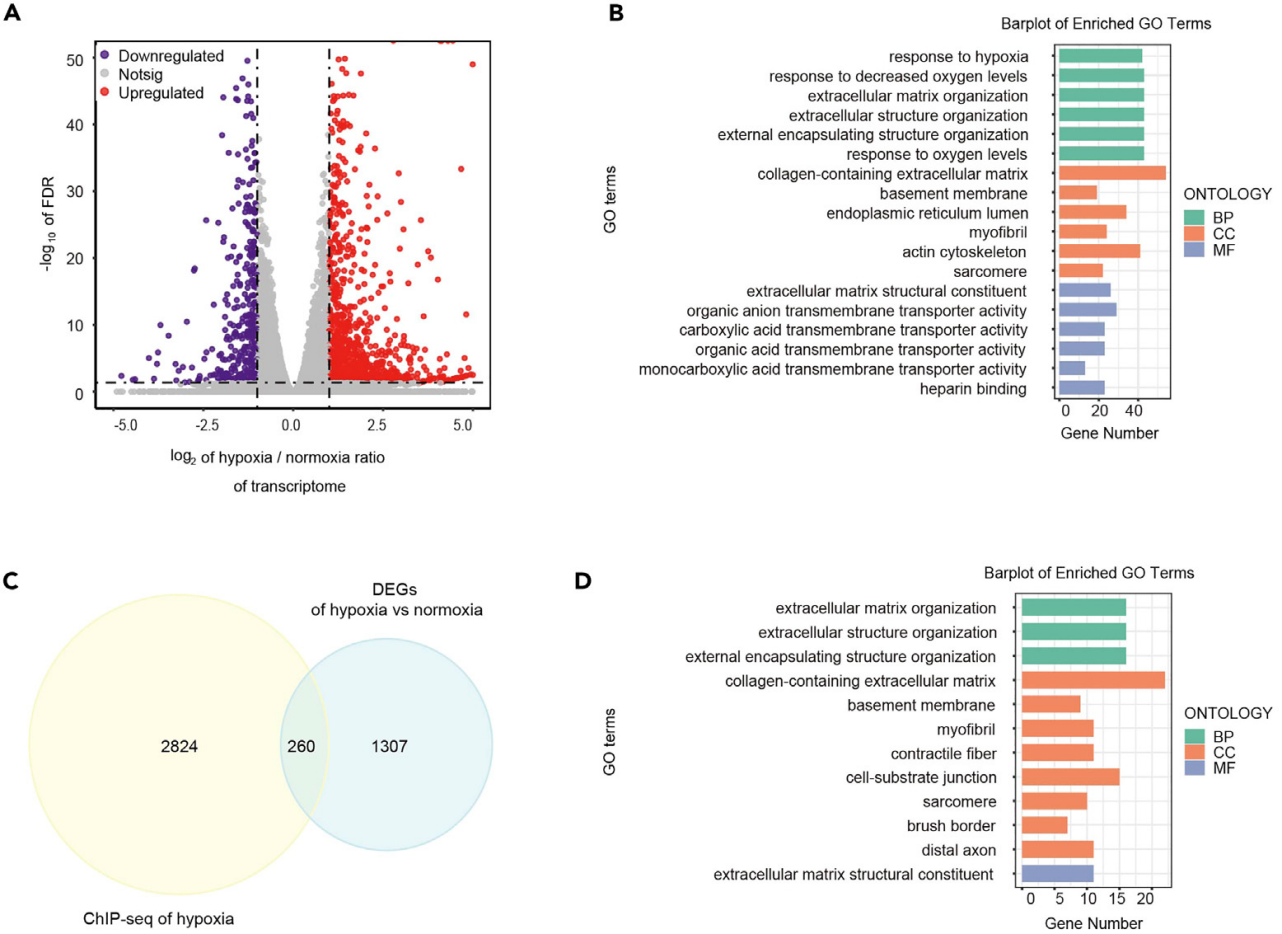
RNA-seq combined with H3K9la MNase ChIP-seq and proteomics analysis in KYSE30 cells cultured in hypoxia (A) Volcano map presents that 1110 mRNAs were upregulate and 402 mRNA were down-regulate in hypoxia group (p adjust<0.05, |Log_2_FC|>1). (B) GO enrichment analysis of differentially expressed genes in KYSE30 cells cultured in normoxia and hypoxia. (C) 260 genes were observed in an overlapped combination of differentially expressed genes and H3K9la binding different peaks in KYSE30 cells cultured in normoxia and hypoxia. (D) GO enrichment analysis of the overlapped genes.

To examine the transcription of target gene by H3K9la, we further performed total RNA-seq analyses of KYES30 cells cultured in 1% O_2_ or 21% O_2_ for 48 h. RNA-seq analysis showed that 1110 mRNAs were upregulate and 402 mRNA were downregulated in hypoxia group (Figures 4A and S4A). GO enrichment analysis of differentially expressed genes (DEGs) revealed that extracellular matrix was significantly influenced by hypoxia, including extracellular matrix organization, extracellular structure organization, extracellular matrix, and so on (Figure 4B). Interestingly, KEGG enrichment also showed that the EDGs were enriched in ECM-receptor interaction and focal adhesion pathway which were related with extracellular matrix (Figure S4B).

**Figure 5:**
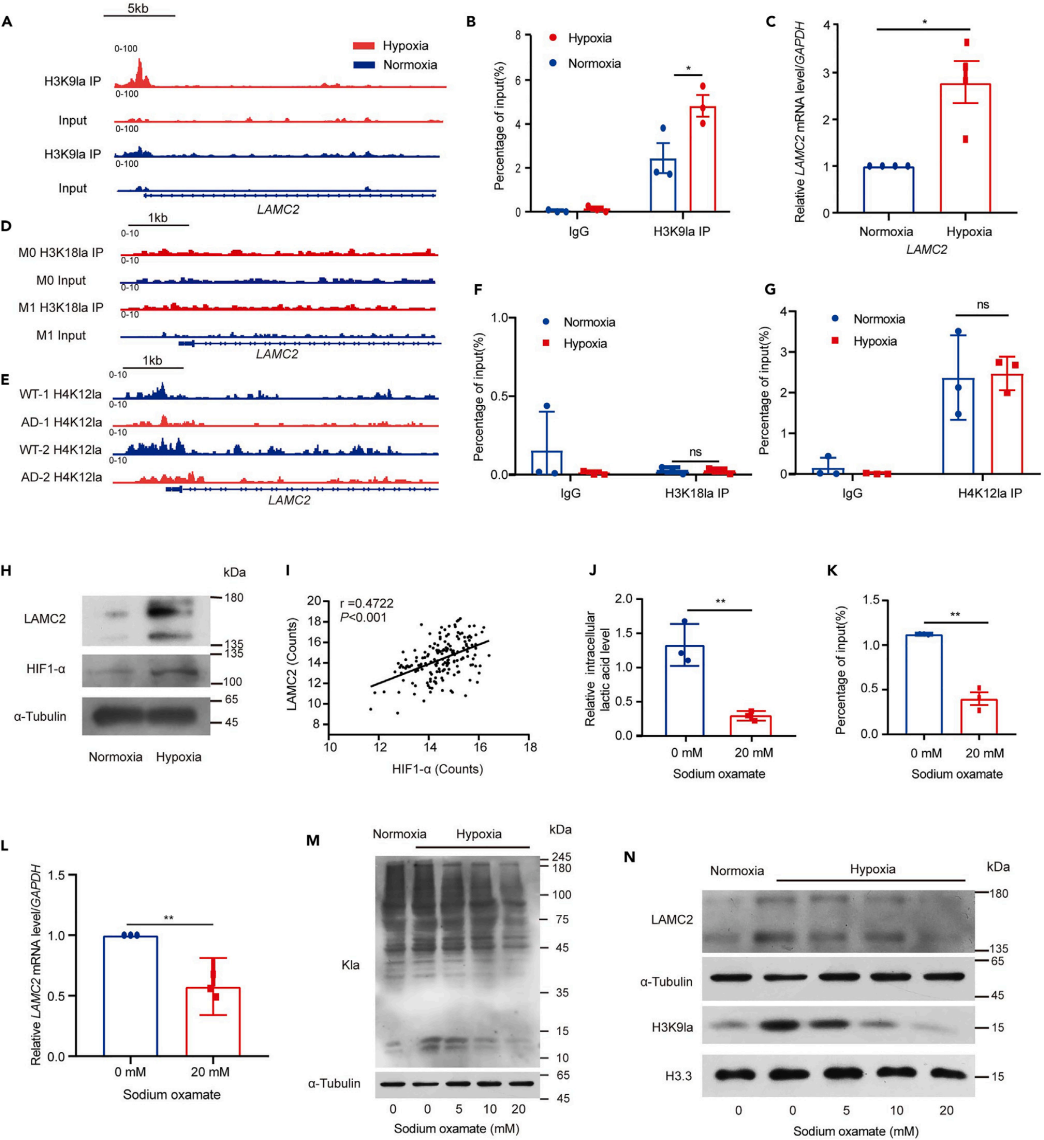
H3K9la influence the expression of *LAMC2* in KYSE30 cells under hypoxia (A) *LAMC2* promoter in the genomic position was identified to enrich in H3K9la peaks when KYSE30 cells cultured in hypoxia. (B) The ChIP-qPCR assays showed that H3K9la levels on *LAMC2* promoter were significantly elevated in KYSE30 cells cultured in hypoxia (*n* = 3 per group). (C) The qPCR assays monitoring expression of the *LAMC2* in KYSE30 cells cultured in hypoxia (*n* = 4 per group). (D) The ChIP-seq showed that H3K18la was not enriched in *LAMC2* promoter in macrophage (GSE115354). (E) The CUT&Tag-seq showed that *LAMC2* promoter in the genomic position was identified to enrich in H4K12la peaks in microglia. (F) The ChIP-qPCR assays showed that H3K18la can’t not enriched in *LAMC2* promoter in KYSE30 cells cultured in hypoxia or normoxia (*n* = 3 per group). (G) The ChIP-qPCR assays showed that H4K12la levels on *LAMC2* promoter were not changed in KYSE30 cells cultured in hypoxia (*n* = 3 per group). (H) Western blotting analysis of LAMC2 in KYSE30 cells in KYSE30 cells cultured in hypoxia or normoxia. (I) TCGA-ESCC data showed that the expression of *LAMC2* were related with *HIF1-α*. (J) Sodium oxamate decreased the intracellular lactic acid in KYSE30 cells (*n* = 3 per group). (K) The ChIP-qPCR assays showed that H3K9la levels on *LAMC2* promoter were decreased in KYSE30 cells stimulated by sodium oxamate in hypoxia (*n* = 3 per group). (L) The qPCR assays monitoring expression of the *LAMC2* in KYSE30 cells stimulated by sodium oxamate in hypoxia (*n* = 3 per group). (M) Pan Kla decreased in KYSE30 cells stimulated by sodium oxamate under hypoxia. (N) H3K9la and LAMC2 were decreased in KYSE30 cells stimulated by sodium oxamate under hypoxia. Statistical significance was analyzed by *Student’s t-test.* Data are mean ± SEM. ∗*p* < 0.05, ∗∗*p* < 0.01, ns indicates no significance.

To determine whether the DEGs induced by H3K9la in hypoxia, we next compared the H3K9la binding sites with the DEGs in RNA-seq analysis and found 260 genes merged in H3K9la binding sites (Figure 4C and Table S2). GO enrichment analysis of these genes showed that extracellular matrix relative pathways were significantly enriched including extracellular matrix organization, collagen-containing extracellular matrix, and extracellular matrix structural constituent (Figure 4D). Meanwhile, reactome pathway showed these genes mostly enriched in extracellular matrix organization and cell junction organization (Figure S4C). Together, these results indicate that H3K9la binding genes in hypoxia may regulate the transcription of extracellular matrix genes in KYSE30 cells. By overlapping the gene sets among MNase ChIPseq and RNA-seq, we found that the *LAMC2* was one of the most significantly increased genes enriched in collagen-containing extracellular matrix in RNA-seq data and its promoter in the genomic position was identified to enrich in H3K9la peaks (Figures 5A and S4D). LAMC2 could interact with other extracellular matrix components, suggesting that H3K9la may be involved in the regulation of the LAMC2 expression in KYSE30 cells.

### H3K9la modulates the expression of LAMC2 in KYSE30 cells under hypoxia

To validate the regulation of H3K9la in the expression of *LAMC2* under hypoxia, we conducted chromatin immunoprecipitation followed by quantitative PCR (ChIP-qPCR) and quantitative real-time PCR (real-time qPCR) to confirm our MNase ChIP-seq and RNA-seq results. The ChIP-qPCR results showed that H3K9la levels on *LAMC2* promoter were significantly elevated in KYSE30 cells cultured in hypoxia (Figure 5B). The qPCR and western blot results both showed that the level of *LAMC2* mRNA and protein were increased in KYSE30 cells cultured in hypoxia (Figures 5C and 5H). To examine if the H3K9la specially binds to gene of *LAMC2* under hypoxia, we analyzed the potential target genes regulated by other histone Kla (H3K18la and H4K12la) through GEO datasets (GEO: GSE188765 and GSE115354), and found that H3K18la is not enriched but H4K12la in the *LAMC2* promoter (Figures 5D and 5E). The ChIP-qPCR analysis further validated the H3K18la result (Figure 5F). However, the enrichment of H4K12la is not altered in *LAMC2* promoter in KYSE30 cells whether hypoxia or normoxia (Figure 5G). These results suggest that hypoxia-induced histone H3K9la prefers to regulate *LAMC2.* Besides, analysis of TCGA-ESCC data suggested that the expression of *LAMC2* were related with *HIF1-α* (Figure 5I).

Next, we used sodium oxamate, which can inhibit cell product lactate (Figure 5I), to treat KYSE30 cells cultured in hypoxia. We found that the Pan Kla and H3K9la were both decreased in KYSE30 cells stimulated by sodium oxamate under hypoxia (Figures 5M and 5N). The ChIP-qPCR results showed that H3K9la levels on *LAMC2* promoter were decreased in KYSE30 cells stimulated by sodium oxamate in hypoxia (Figure 5L). Meanwhile, the qPCR and western blot results showed that the level of *LAMC2* mRNA and protein were decreased in KYSE30 cells cultured in hypoxia (Figures 5M and 5N). Together, we conclude that H3K9la regulates the expression of *LAMC2* in KYSE30 cells under hypoxia.

### LAMC2 promotes ESCC migration and invasion *in vitro* and metastasis *in vivo*

**Figure 6:**
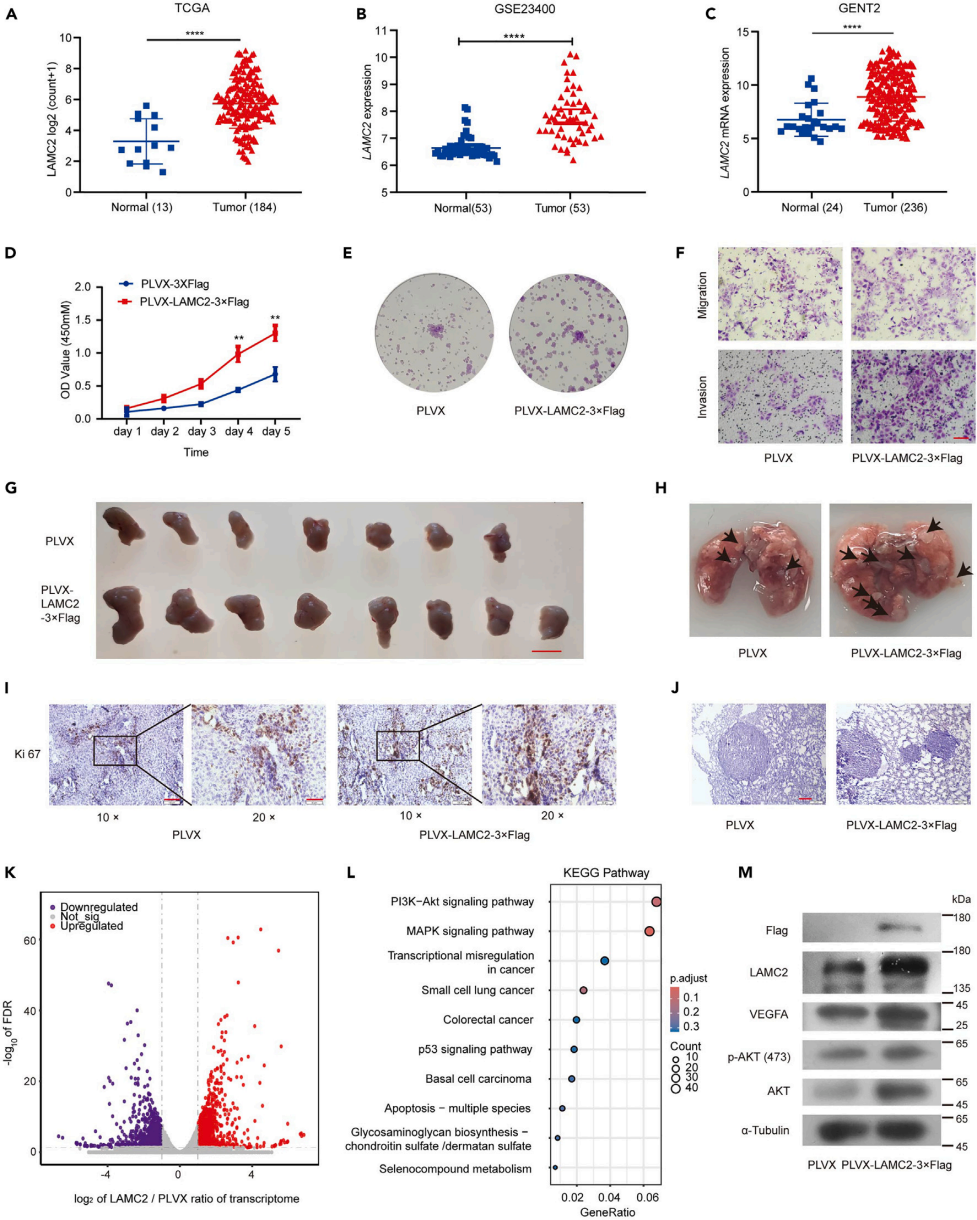
LAMC2 plays an oncogenic role in ESCC (A–C) TCGA, GSEA, GENT2 data showed that *LAMC2* mRNA levels was highly expressed in ESCC tumors compared to the normal controls. (D) CCK-8 assay showed that overexpress LAMC2 promoted the cell viability (*n* = 3 per group). (E) Colony formation assay of KYSE30 cells with overexpression of LAMC2. (F) Transwell assays show the migratory and invasive abilities of KYSE30 cells with overexpression of LAMC2. The scale bar shows 100 μm. (G) Overexpressed LAMC2 and control KYSE30 cells were subcutaneously implanted into mice. The tumor sizes were continuously recorded to draw tumor growth curves. The scale bar shows 1 cm. (H) The number of metastatic tumor foci in lung. (I) Representative images of Ki-67 staining of tumor sections in xenograft mouse models. The scale bar shows 100 μm in figure 10 × and 50 μm in figure 20 ×. (J) Hematoxylin-eosin staining of metastatic tumor foci in lung. The scale bar shows 200 μm. (K) Volcano map presents that 1071 mRNAs were up-regulate and 937 mRNA were down-regulate in LAMC2 overexpressed KYSE30 cells (p adjust<0.05, |Log_2_FC|>1). (L) KEGG enrichment analysis of the DEGs. (M) Expression of VEGFA, AKT and *p*-AKT in overexpressed LAMC2 KYSE30 cells. Statistical significance was analyzed by *Student’s t-test*. Data are mean ± SEM. ∗*p* < 0.05, ∗∗*p* < 0.01.

To reveal the expression of *LAMC2* in ESCC tissues, we analyzed the available data from GEPIA2 (GEPIA 2: cancer-pku.cn), GENT2 (GENT2: http://gent2.appex.kr/gent2/), and GEO datasets (GEO: GSE161533, GSE38129, and GSE23400), and found that *LAMC2* mRNA level was highly expressed in ESCC tumors compared to the normal controls (Figures 6A–6C, S5A, and S5B). In addition, LAMC2 level was tested in immortalized normal esophageal epithelium and ESCC cells lines by using western blot assay. Our results showed that LAMC2 was highly expressed in these ESCC cell lines compared with NE3 cell line (Figure S5C). To investigate the roles of LAMC2 in ESCC, an overexpressed LAMC2 in KYSE30 cell was established and further performed a series of evaluations for tumor behaviors. CCK8 assay showed that overexpression of LAMC2 significantly promoted the cell viability in KYSE30 cells (Figure 6D). The colony formation assay revealed that overexpression of LAMC2 increased the formation rate in KYSE30 cells (Figures 6E and S5D). Additionally, transwell assay showed that overexpress of LAMC2 elevated KYSE30 cells migration and invasion (Figures 6F and S5E). Finally, wound-healing assays demonstrated that overexpression of LAMC2 promoted cell motility (Figures S5F and S5G). These results reminded that LAMC2 may play a promoting role on ESCC progression *in vitro*. Furthermore, the xenograft mouse models were used to confirm our finding *in vivo*. Consistently, overexpressed of LAMC2 resulted in a significant increase in the formation and lung metastasis of ESCC tumor *in vivo* (Figures 6G–6J and S5H–S5K). Meanwhile, overexpressed of LAMC2 markedly advanced Ki-67 activity in tumor tissues compared to control (Figure 6I). Collectively, both *in vitro* and *in vivo* results suggest that LAMC2 promotes the malignant phenotype of ESCC cells.

### LAMC2 activates the PI3K/Akt signaling pathway to influence the expression of VEGFA

To dissect the mechanism underlying LAMC2-promoted ESCC tumor progression, RNA-seq analysis were performed and showed that 1071 mRNAs were upregulated and 937 mRNAs were downregulated in KYSE30 cells upon LAMC2 overexpression (Figures 6K and S5M). The KEGG pathway enrichment analysis showed that the DEGs were mostly enriched in “PI3K-Akt signaling pathway” and “MAPK signaling pathway” (Figure 6L). We found that the *VEGFA*, one of downstream gene in PI3K-Akt signaling pathway, is signally upregulated in overexpressed LAMC2 KYSE30 cells (Figure S5N). Given that the AKT is a key to PI3K-Akt signaling pathway,^30^ we further validated that overexpressed LAMC2 would increase the *p*-AKT and VEGFA level (Figure 6M). Meanwhile, overexpressed of LAMC2 increased the level of VEGFA and *p*-AKT in xenograft tumor tissues (Figure S5L). Besides, analysis of TCGA-ESCC data suggest that the expression of *LAMC2* is related with *VEGFA* level (Figure S5O). Collectively, LAMC2 may have influence on the expression of *VEGFA*, after activating the PI3K/Akt signaling pathway.

**Figure 7:**
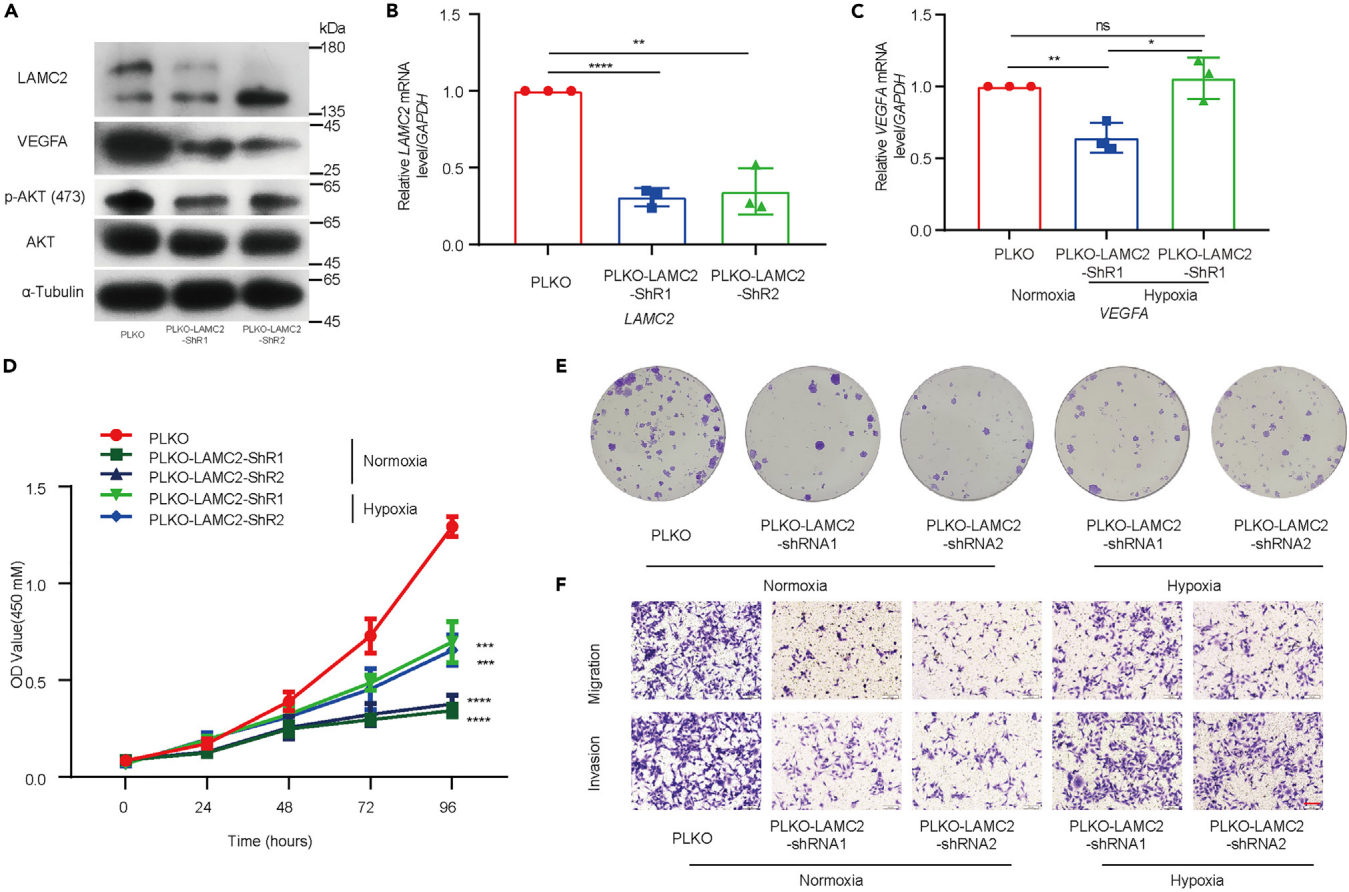
LAMC2 activates the PI3K/Akt signaling pathway to influence the expression of VEGFA (A) Expression of VEGFA, AKT, and *p*-AKT in knockdown LAMC2 KYSE30 cells. (B) The expression of the LAMC2 in knockdown LAMC2 KYSE30 cells (*n* = 3 per group). (C) The expression of the VEGFA in knockdown LAMC2 KYSE30 cells cultured in hypoxia (*n* = 3 per group). (D) CCK-8 assay showed that the cell viability of knockdown LAMC2 KYSE30 cells cultured in hypoxia (*n* = 3 per group). (E) Colony formation assay of KYSE30 cells with knockdown of LAMC2 in hypoxia (*n* = 3 per group). (F) Transwell assay show the migratory and invasive abilities of KYSE30 cells with knockdown of LAMC2 in hypoxia. The scale bar shows 100 μm (*n* = 5 per group). Statistical significance was analyzed by *Student’s t-test*. Data are mean ± SEM. ∗*p* < 0.05, ∗∗*p* < 0.01., ∗∗∗*p* < 0.001, ∗∗∗∗*p* < 0.0001, ns indicates no significance.

In order to explore the role of the LAMC2 in hypoxia environment, we established two knockdown of LAMC2 in KYSE30 cells (Figures 7A and 7B) and further performed a series of experiments in hypoxia and normoxia environments. qPCR assay showed that knockdown of LAMC2 decreased the expression of *VEGFA* mRNA and hypoxia will alleviate this phenomenon (Figure 7C). CCK8 assay showed that hypoxia could improve the cell viability in KYSE30 cells which was suppressed by knockdown LAMC2 (Figure 7D). The colony formation assay revealed that knocking down of LAMC2 decreased the formation rate in KYSE30 cells while hypoxia could improve the formation rate of LAMC2 knocking down cells (Figures 7E and S6C). Additionally, transwell assay showed that knocking down of LAMC2 decreased KYSE30 cells migration and invasion, conversely, hypoxia could elevate these cells migration and invasion (Figures 7F and S6D). Finally, wound-healing assays demonstrated that attenuation of LAMC2 expression decreased cell motility, while hypoxia could reverse this phenomenon (Figures S6A and S6B). Collectively, these results indicate that hypoxia could reverse the tumor suppressor which were caused by attenuated LAMC2 expression.

## Discussion

Tumor cells consume a lot of oxygen and nutrient utilization to maintain the faster replication and higher biosynthesis, but the solid tumors always growth in hypoxia environment.^31^^,^^32^^,^^33^^,^^34^ Accumulating evidence shows that normal energy metabolism is significantly influenced under hypoxia microenvironment, but tumor cells could get enough energy by metabolic reprogramming.^35^ However, tumor cells get energy mainly through glycolysis, even under sufficient oxygen environment.^36^ Glycolysis not only provide abundant ATP for tumor cells to maintain different kind of biological process, but also provides lactate and nicotinamide adenine dinucleotide hydrogen (NADH).^37^ People used to think that lactate is a metabolic waste product in cells, because lactate is produced in intense exercised muscle and ischemic tissues.^38^ In recent years, mounting evidence suggests that lactate also serves as a major circulating carbohydrate fuel.^39^^,^^40^ In this study, we found that cell metabolism was changed obviously when KYSE30 cells was cultured under hypoxia; the glucose uptake and lactate production of KYSE30 cells was significantly increased. Meanwhile, we showed that hypoxia obviously promoted the malignant phenotype of KYSE30 cells.

Overwhelming evidence shows that histone PTMs such as Kac, Kme, Khib, and Ksuc are key regulators for gene expression.^41^^,^^42^^,^^43^^,^^44^ Histone lactylation induced by hypoxia is a newly identified type of PTM, involving in the epigenetic regulation.^45^^,^^46^ Growing evidence suggested that histone lactylation modulates multiple biological processes including macrophage differentiation, oncogenesis in ocular melanoma and progress of Alzheimer’s disease.^5^^,^^47^^,^^48^ Recent studies suggest histone lactylation can be catalyzed by the acetyltransferase p300 and cleaved by HDAC1–3 and SIRT1–3.^49^ While our recent studies have demonstrated that the HBO1 can catalyze lysine lactylation and mediates histone H3K9la to regulate gene transcription including *LAMC2*^50^ In the present study, we found that global level of Kla was elevated in ESCC under hypoxia. And then, we characterized that landscape of histone Kla by combining Kla affinity enrichment with SILAC-based proteomics analysis, mapping 72 Kla sites on histones. By combing with bioinformatics analysis, we further revealed the key histone Kla sites triggered by hypoxia and validated significant increase of H3K9la level. To investigate the attendant epigenetic modulatory impacts of H3K9la in KYSE30 cells cultured under hypoxia, we used MNase ChIP-seq to identify candidate genes and found the enrichment of extracellular matrix and cell-junction related pathway under hypoxia, suggesting that H3K9la play a key role in migration and invasion under hypoxia.

Altered histone PTMs can influence the transcriptional activation or inhibition of target genes in cancer, where these changes may act as oncogenic drivers. To identify the transcriptional of potential target genes regulated by H3K9la, we combined the RNA-seq and NMase-seq to reveal 260 (202 up and 58 down in RNA-seq) genes merged. Reactome pathway and GO analysis of these genes showed that significant extracellular matrix relative pathways were significant enrich again. These results indicated H3K9la may be involved in the regulation of the transcription of extracellular matrix and cell-junction related genes in KYSE30 cells under hypoxia. By multi-omic analysis, we identify *LAMC2* as a potential specific downstream target of histone H3K9la.

It is well known that hypoxia would influence the metabolic reprogramming and increase the expression of glycolytic enzymes. Growing research has also showed that histone lactylation links to gene expression related to glucose metabolism.^47^ For example, histone H4K12la modulates the expression of glycolytic enzymes in AD microglia.^48^ However, our NMase-seq results did not show a significant association between H3K9la and glycolytic. These insights imply that different histone lactylation sites may regulate different genes, and hypoxia-triggered histone lactylation regulated genes might be different from histone lactylation in other system. Here, we found that the *LAMC2* promoter has a marked enrich in H3K9la and H3K9la activated the transcription of *LAMC2* in KYSE30 cells. By using sodium oxamate to inhibit the production of lactate in KYSE30 cells cultured in hypoxia, we found H3K9la levels on *LAMC2* promoter were decreased, suggesting that H3K9la is likely to act as a bridge between hypoxia and *LAMC2*.

Previous research showed that LAMC2 has been linked to various cancers, such as lung cancer, gastric cancer, and pancreatic cancer.^51^^,^^52^^,^^53^^,^^54^ TCGA data also showed that *LAMC2* was significantly increased with multiple tumor tissues. As an important component of extracellular matrix organization and cell junction, LAMC2 would influence the tumorigenesis. Herein, we identified the expression of LAMC2 was significantly increased under hypoxia, and showed that LAMC2 promoted tumor progression *in vitro* and *in vivo*, which was consistent with the role of hypoxia. And LAMC2 activates the PI3K/Akt signaling pathway to influence the expression of VEGFA.

In summary, we identified the histone Kla landscape under hypoxia in ESCC and showed that histone H3K9la could activate the *LAMC2* transcription to promote the tumor progression. Our study reveals a hypoxia-induced, Kla-dependent molecular mechanism for the regulation of transcription, providing a different insight into hypoxia promote the tumor behaviors.

### Limitations of the study

A limitation to our studies is that we focused on hypoxia promotes histone H3K9 lactylation to enhance ESCC progression; however, hypoxia is a complex biological process in tumors progression. HIF1-α has been known as a transcription factor which would be more stabilize in hypoxia environment and promote tumors progression. Notably, hypoxia induces metabolic reprogramming, in our studies we only focused on glucose uptake and lactate production. It is well known that cell could use lipid and amino acid to provide energy. These metabolites can also affect the histone modifications such as histone acetylation, succinylation, and methylation. Another limitation is that we only focus on one histone lysine lactylation site in our studies. We found hypoxia induces a general increase of histone Kla level, and we could not ignore the function of these sites. Finally, clinical samples and clinical information to validate one-way regression on H3K9la and LAMC2, as well as prognostic information on these two proteins are lack in this study, which limits its application for clinical guidance.

## STAR★Methods

### Key resources table

**Table:** 

REAGENT or RESOURCE	SOURCE	IDENTIFIER
**Antibodies**		

Alpha Tubulin Polyclonal antibody	Proteintech	Cat No. 11224-1-AP; RRID: AB_2210206
Anti-L-Lactyl-Histone H3 (Lys9) Rabbit mAb	PTMBIO	Cat: PTM-1419RM; RRID: AB_3076695
Anti-L-Lactyl-Histone H3 (Lys18) Rabbit mAb	PTMBIO	Cat: PTM-1406RM; RRID: AB_2909438
Anti-L-Lactyl-Histone H4 (Lys5) Rabbit pAb	PTMBIO	Cat: PTM-1407; RRID: AB_3096309
Anti-L-Lactyl-Histone H4 (Lys8) Rabbit mAb	PTMBIO	Cat: PTM-1415RM; RRID: AB_3101829
Anti-H4K12la Rabbit pAb	PTMBIO	Cat: PTM-1411; RRID: AB_2941896
Anti-L-Lactyl-Histone H4 (Lys16) Rabbit mAb	PTMBIO	Cat: PTM-1417RM; RRID: AB_3101830
Anti-Histone H3.3 Rabbit mAb	PTMBIO	Cat: PTM-7294; RRID: AB_3101831
Anti-L-Lactyl Lysine Rabbit pAb	PTMBIO	Cat: PTM-1401; RRID: AB_2868521
LAMC2 Polyclonal antibody	Proteintech	Cat No. 19698-1-AP; RRID: AB_10644139
VEGFA Polyclonal antibody	Proteintech	Cat No. 19003-1-AP; RRID: AB_2212657
DYKDDDDK tag Monoclonal antibody	Proteintech	Cat No. 66008-4-Ig; RRID: AB_2918475
HIF-1 alpha Polyclonal antibody	Proteintech	Cat No. 20960-1-AP; RRID: AB_10732601
LDHA Rabbit pAb	abclonal	Cat: A1146; RRID: AB_2758572
Ki67 Rabbit pAb	abclonal	Cat: A11390; RRID: AB_2758539
AKT Monoclonal antibody	Proteintech	Cat No. 60203-2-Ig; RRID: AB_10912803
Phospho-AKT (Ser473) Recombinant antibody	Proteintech	Cat No. 80455-1-RR; RRID: AB_2918892
Anti-L-lactyllysine antibody conjugated agarose beads	PTMBIO	Cat: PTM-1404; RRID: AB_3101832

**Bacterial and virus strains**		

DH5α Competent Cells	Sangon Biotech	Cat: B528413-0100

**Chemicals, peptides, and recombinant proteins**		

Trypsin	Beijin life proteomic	Cat: 9002-07-7
Deacetylase Inhibitor Cocktail 100 ×	Beyotime	Cat: P1113
Trizol	Invitrogen	Cat: 15596026
2 - NBDG	MedChemExpress	Cat: 186689-07-6
MNase	Merck	Cat: 9013-53-0
2 × RealStar Fast SYBR qPCR Mix	GenStar	Cat: A301-10
DMEM for SILAC	Thermo Scientific™	Cat: A33822
Fetal Bovine Serum Dialyzed	ThermoFisher	Cat: 30067334
L-Argnie- HCl	ThermoFisher	Cat: 88427
L-^13^C_6_-lysine-2 × HCl	ThermoFisher	Cat: 88431
L-lysine-2 × HCl	ThermoFisher	Cat: 88429
Fetal Bovine Serum	VivaCell	Cat: C2910-0500
Roswell Park Memorial Institute (RPMI) 1640	ThermoFisher	Cat: C3001-0500
Penicillin-Streptomycin Solution, 100×	Beyotime	Cat: C0222
Defined Keratinocyte-SFM a serum-free medium	Gibco	Cat: MEPI500CA
EpiLife	Gibco	Cat: 10744019
RIPA Lysis Buffer	Beyotime	Cat: P0013B
PMSF	Beyotime	Cat: ST505
C18 ZipTips	Millipore Corp	Cat: ZTC18M096

**Critical commercial assays**		

Pierce™ BCA Protein Assay Kit	Thermo Scientific™	Cat: 23227
CheKine™ Micro Lactate Assay Kit	Abbkine	Cat: KTB1100
DAB Kit	Solarbio	Cat: DA1015
Anti-L-lactyllysine antibody conjugated agarose beads	PTM	PTM-1014K

**Deposited data**		

MS/MS analysis for protein quantification	This paper	ProteomeXchange Consortium: PXD048995.
MNase ChIP-seq and RNA-seq data	This paper	Gene Expression Omnibus (GEO): GSE255352

**Experimental models: Cell lines**		

KYSE 30	Pricella Life	Cat: CL-0577
KYSE 140	FengHuiShengWu	Cat: CL0437
KYSE 150	Pricella Life	Cat: CL-0638
KYSE 450	FengHuiShengWu	Cat: CL0606
NE3	BLUEFBIO™	Cat: BFN607200102
HEK-293T	ATCC	Cat: CRL-11268

**Experimental models: Organisms/strains**		

BALB/c nude	Beijing Vital River Laboratory Animal Technology Co., Ltd	https://buy.vitalriver.com/commodity/detail?productId=402883af7e23dcd2017e23dd211e0adb

**Oligonucleotides**		

*LAMC2* ChIP3-For 5’ GTGGAGAGGACCCTGTTGTG 3’	tsingke	HPLC-purified
*LAMC2* ChIP3-Rev 5’ CCAGGGCAGATTCACAGAGG 3’	tsingke	HPLC-purified
*LAMC2* qPCR-For 5’ GACAAACTGGTAATGGATTCCGC 3’	tsingke	HPLC-purified
*LAMC2* qPCR-Rev 5’ TTCTCTGTGCCGGTAAAAGCC 3’	tsingke	HPLC-purified
*GAPDH*-qPCR-For 5’ GCACCGTCAAGGCTGAGAAC 3’	tsingke	HPLC-purified
*GAPDH*-qPCR-Rev 5' TGGTGAAGACGCCAGTGGA 3'	tsingke	HPLC-purified
*LAMC2*-shRNA1 5’ GCCCTGCAATTGTAACTCCAA 3’	tsingke	HPLC-purified
*LAMC2*-shRNA2 5’ GCTCACCAAGACTTACACATT 3’	tsingke	HPLC-purified
5’ AGGGCAGAATCATCACGAAGT 3’ *VEGFA*-qPCR-For	tsingke	HPLC-purified
5’ AGGGTCTCGATTGGATGGCA 3’ *VEGFA*-qPCR-Rev	tsingke	HPLC-purified

**Recombinant DNA**		

Plasmid: plvx-puro	This paper	N/A
Plasmid: Plko.1-puro	This paper	N/A

**Software and algorithms**		

R	https://www.r-project.org	N/A
DESeq2	https://github.com/mikelove	N/A
GraphPad Prism 8	www.graphpad.com	N/A
MaxQuant (v.1.5.5.1)	https://www.maxquant.org/	N/A

### Resource availability

#### Lead contact

Further information and requests for resources and reagents should be directed to and will be fulfilled by the lead contact, Kai Zhang (kzhang@tmu.edu.cn).

#### Materials availability

This study did not generate new unique reagents.

#### Data and code availability

The data supporting the findings of this study are available within the article and its supplemental information. The mass spectrometry proteomics data have been deposited to the ProteomeXchange Consortium (http://proteomecentral.proteomexchange.org) via the iProX partner repository with the dataset identifier PXD048995. The MNase ChIP-seq and RNA-seq data generated in this study have been deposited in the Gene Expression Omnibus (GEO) repository under accession code GSE255352.

This study did not generate original code.

Any additional information required to reanalyze the data reported in this paper is available from the lead contact upon request.

### Experimental model and study participant details

#### Cell lines

All cells were cultured in RPMI-1640 medium supplemented with 10% FBS and 1% antibiotics at 37°C in a humidified atmosphere with 5% or a humidified atmosphere with 5% or 1% CO_2_. KYSE30 cells were grown in lysine and arginine deficient RPMI-1640, supplemented with 10% dialyzed fetal bovine serum 100 U/ml of penicillin and streptomycin, 2 mM ^12^C_6_ -L-lysine arginine, and 2 mM^13^C_6_ -L-lysine or ^12^C_6_ -L-lysine.

#### Animals

Eight-week-old male mouses were purchased from Beijing Vital River Laboratory Animal Technology Company. Mouse experiments were approved by Tianjin Medical University Animal Care and Use Committee. Mouses were under a circadian cycle of light: dark = 12: 12 h. All procedures and experiments were approved by the Laboratory Animal Management and Use Committee (IACUC) of Tianjin Medical University. (Doc. No): TMUaMEC2024018.

### Method details

#### SILAC labeling and cell culture

ESCC cell lines were cultured in RPMI-1640 medium and NE3 cells were cultured in Defined Keratinocyte-SFM a serum-free medium: EpiLifeis (1:1) and supplemented with 10% FBS and 1% antibiotics at 37°Cin a humidified atmosphere with 5% CO_2_ and 21% or 1% O_2_. The construction of LAMC2 stable knockdown or overexpress in KYSE30 cells was performed according to the PLKO.1 and PLVX protocol. KYSE30 cells were grown in lysine and arginine deficient RPMI-1640, supplemented with 10% dialyzed fetal bovine serum 100 U/ml of penicillin and streptomycin, 2 mM ^12^C_6_ -L- arginine, and 2 mM^13^C_6_ -L-lysine or ^12^C_6_ -L-lysine. Each population were grown for at least five population doublings. And the labeling efficiency was greater than 98%.

#### Histone extraction

Histones from cells were extracted using an acid-extraction protocol.^55^ Cells were collected in lysis buffer (PBS with 1%NP-40, Deacetylase Inhibitor Cocktail 100 x, 1 Mm Protease Inhibitor Cocktail and 1 mM PMSF) and revolve slowly for 15 min. The nuclei were collected by centrifugation at 1000 g for 15 min at 4°C then resuspended in 0.2 M H_2_SO_4_, gentle rotation over night at 4°C. The mixture was centrifuged at 1 6000 g for 15 min at 4°C. The supernatants were collected and mixed with 25% (final concentration) trichloroacetic acid on ice. The mix static precipitation 30 min. The liquids were centrifuged at 16000 g for 30 min at 4°C. After acetone washing thrice and drying, the histone was dissolved by 100mM NH_4_HCO_3_ for next experiment.

#### SILAC-MS sample preparation and immunoaffinity enrichment

Collected ^13^C_6_ -L-lysine and ^12^C_6_-L-lysine labeling histone as Figure 2B. Equal amounts of histones form KYSE30 cells cultured in 1% O_2_ or 21% O_2_ for 48 h were mixed and overnight digestion by trypsin (trypsin: protein ratio, 1:50). 10% trifluoroacetic acid (TFA) terminate digestion and products were desalted by SepPak C 18 cartridges and dried. Peptides immunoaffinity enrichment was using an the 20 μl anti-lactyllysine antibody-conjugated protein A agarose beads were used for 1 mg histones. Bound peptides were eluted three times with 1% trifluoroacetic acid. Finally, the eluate buffer was dried and cleaned with gentle rotation C18 Zip Tips before HPLC–MS/MS analysis.

#### HPLC-MS/MS analysis for Kla

Enriched peptides were analyzed by HPLC-MS/MS. The method was described as previously.^56^ In detail, combination of -45 V /- 60 V FAIMS CVs (compensation voltage) were set to run DDA mode for 1 s cycle to build a big cycle of 3 s.

#### Database search and data filter criteria for Kla

The database search and filter criteria were performed as described previously.^56^ Raw data were searched by MaxQuant (v.1.5.5.1) with UniProt human protein database (Proteome ID: UP000474145) and an overall false discovery rate for peptides of less than 1%. Peptide sequences searching was set as trypsin specificity, six missed cleavages for maximum and seven for minimal. Lactylation, methylation, dimethylation, trimethylation, acetylation on lysine, oxidation of methionine and acetylation on the peptide N terminus were fixed as variable modifications. Mass tolerances were set at ±10 ppm for precursor ions and ±0.02 Da for MS/MS, and lactylated peptides with a score <40 and localization probability <0.75 were further excluded.

#### Migration and invasion assays

For the migration assay, 2×10^4^ cells were resuspended by serum-free RPMI-1640 media and then seeded into the upper chamber of well (8 μm pore size). For the invasion assay Matrigel was diluted with serum-free RPMI-1640 media and then plated into the upper chamber of well. 4x10^6^ cells were resuspended by serum-free RPMI-1640 media and then seeded into the upper chamber of well. RPMI-1640 media 20% FBS was added into the down well. Cells cultured 36-48 h, fixed with 4% paraformaldehyde and stained with crystal violet.

#### MNase ChIP-seq

The MNase ChIP-seq was performed using a gold-standard’ ChIP protocol.^57^^,^^58^^,^^59^ In brief, 5×10^6^ cells were fixed with 1% formaldehyde at RT for 10 minutes. Crosslinking was stopped by adding 150 mM of glycine for 5 minutes at RT. Cells were washed twice and collected by douncing buffer (10 mM Tris-HCl, pH 7.5, 4 mM MgCl_2_, 1 mM CaCl_2_ and protease inhibitor cocktail). Chromatin was digested in 1 ul MNase at 37°C for 20 min. Reaction was quenched by 0.5 M EDTA. Immunoprecipitation was performed using 1 μl of H3K9la antibody overnight at overnight at 4°C. After elution and reversed crosslinking, samples were treated with RNase A for 30 minutes at 37°C and Proteinase K for 1 h at 55°C. DNA was purified by phenol chloroform and 5 ng of raw ChIP material was processed for library construction. For ChIP sequencing (ChIP-seq), sequenced on the Illumina NovaSeq platform. Quality control was performed by FastQC software. Clean reads were aligned to the human reference genome (hg 38) by bowtie 2 ChIP-seq peaks were detected by the peak-finding algorithm MACS2. The purified DNA also could be used for ChIP-qPCR.

#### RNA sequencing and quantitative real-time PCR

Total RNA was isolated from KYSE30 cells using TRIzol reagent RNA-seq was performed on the Illumina NovaSeq platform. Differentially expressed genes were calculated by DESeq2. The P value corrected by false discovery rate (FDR) was less than 0.05 and the absolute of fold change more than 2 were considered as significant differential expression. GO and KEGG Pathway analysis were performed for differentially expressed genes. 1 ug RNA was reverse transcribed to cDNA and Quantitative PCR was performed using SYBR Green Mix. The relative expression was normalized to GAPDH via the 2^-ΔΔct^ method.

#### Western blot

Protein concentration was determined using BCA Protein Assay Kit. The protein samples were separated by SDS-PAGE, then transferred to nitrocellulose membrane. The membranes were blocked by 5% nonfat milk and incubated with different primary antibodies overnight at 4°C. Antibodies used were H3, H3K9la, H3K18la, H4K5la, H4K8la, H4K12la, H4K16la, α-Tubulin, HIF1-α and LDHA. The membranes were incubated with secondary antibody HRP-conjugated goat anti-mouse or goat anti-rabbit for 2 h at room temperature. The immunoblots were processed with Chemiluminescent Assay Kit.

#### Colony formation assay

KYSE 30 cells were seeded into 12-well plates at 1000 cells/well. Cells were cultured for 7 days and replaced fresh medium every 3 days. Cells were washed twice with PBS and fixed with 4% paraformaldehyde for 15 min, then stain with 0.1% crystal violet solution at room temperature for 10 min. Washed away the dye solution with PBS, took photos and counted the number of cell colonies.

#### Wound healing

The cells were seeded into 6-well plates. When confluence was reached, cell monolayers were scraped using the pipet tip and washed to remove the floating cells with PBS. Then cells were maintained in serum-free medium and Photos were taken during the subsequent 24 h to monitor scratch closure. The results were analyzed by Image J.

#### Immunohistochemistry

The tumors were embedded in paraffin, sectioned and subjected to deparaffinization and rehydration to facilitate antigen retrieval. Sections are then permeabilized and blocked to prevent non-specific antibody binding. The slides were incubated with anti-Ki67 antibodies overnight at 4°C, and HRP-conjugated secondary antibody for 30 minutes. DAB Kit were applied to visualize the antigen-antibody complex.

#### Animal studies

Mouse experiments were approved by Tianjin Medical University Animal Care and Use Committee. Eight-week-old male nude mice were used. 1x10^6^ stable overexpressed LAMC2 KYSE30 cells and PLVX KYSE30 cells were inject into subcutaneous of nude mice (n=4). And we measured the tumor volume three times a week. The tumor volume was length diameter × (width diameter)^2^ × 1/2. For *in vivo* lung metastasis model, 2 × 10^6^ stably KYSE30-PLVX-LAMC2-3×Flag cells and KYSE30-PLVX cells were injected into tail vein of nude mice (n = 4). After 2 months, mice were sacrificed and metastatic lung tumors were analyzed.

#### The detection of intracellular and secreted lactate

Cell culture mediums were collected and executed highly speed centrifugation for 15 min, then used Micro Lactate Assay Kit to measure the concentration of the secreted lactate. Cells were collected in Lactate Assay Buffer and ultrasonicated for 5 min (power 20%, 3 s on and 7 s off). The supernatants were further collected by centrifugation at 12000 g for 5 min at 4°C. Then used Micro Lactate Assay Kit to measure the concentration of the intracellular lactate. Protein concentration of supernatants was detected by using BCA Assay Kit for adjusting the intracellular lactate concentrations from different samples.

### Quantification and statistical analysis

All data were processed and analyzed using Excel and GraphPad prism (Version 8.0.1, http://www.graphpad-prism.cn/) by unpaired two-tailed Student’s t test, one-way-ANOVA and two-way ANOVA. All experiments were repeated at least twice with three replicates involved. Statistical significance is indicated with asterisks as follows: ns, no significance; ∗, *P*< 0.05; ∗∗, *P* < 0.01; ∗∗∗, *P* < 0.001; ∗∗∗∗, *P* < 0.0001.

## Supplemental information

Document S1. Figures S1–S6

Table S1. 72 Kla sites identified on histone proteins, related to Figure 2

Table S2. 260 genes merged from RNA-seq and NMase-seq, related to Figure 4
