# Supplementary material for: Hypoxia promotes histone H3K9 lactylation to enhance LAMC2 transcription in esophageal squamous cell carcinoma
Source: iScience. 2024 Jun 5;27(7):110188. doi: 10.1016/j.isci.2024.110188 (PMC11233973; doi:10.1016/j.isci.2024.110188)
Supplement: Document S1. Figures S1–S6 [file mmc1.pdf]

iScience, Volume *27*

Supplemental information

Hypoxia promotes histone H3K9 lactylation to enhance *LAMC2* transcription in esophageal squamous cell carcinoma

Yong Zang, Aiyuan Wang, Jianji Zhang, Mingxin Xia, Zixin Jiang, Bona Jia, Congcong Lu, Chen Chen, Siyu Wang, Yingao Zhang, Chen Wang, Xinyi Cao, Ziping Niu, Chaoran He, Xue Bai, Shanshan Tian, Guijin Zhai, Hailong Cao, Yupeng Chen, and Kai Zhang

Supplemental Information

Hypoxia promotes histone H3K9 lactylation to enhance *LAMC2* transcription in esophageal squamous cell carcinoma

Supplemental Figures

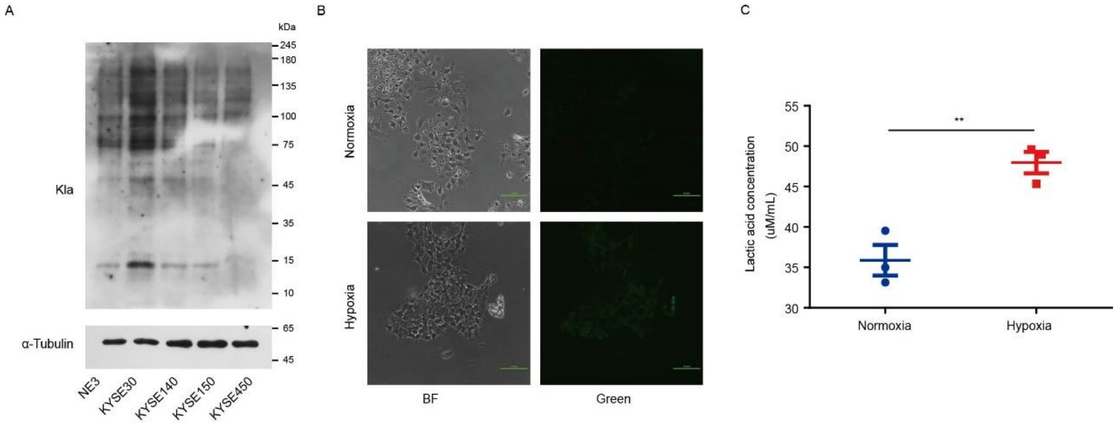

Figure S1. Hypoxia promoted ESCC cells progression and influenced metabolic reprogramming, related to Figure 1.

(A) The lactylation of normal esophageal epithelium and ESCC cell lines.

(B) The secreted lactic acid levels were increased when KYSE30 cells cultured in hypoxia environment. Bar = 100 μm.

(C) Hypoxia significantly increased glucose uptake in KYSE30 cells (n = 3 per group).

Statistical significance was analyzed by *Student's t-test.* Data are mean ± SEM. $**P < 0.01$.

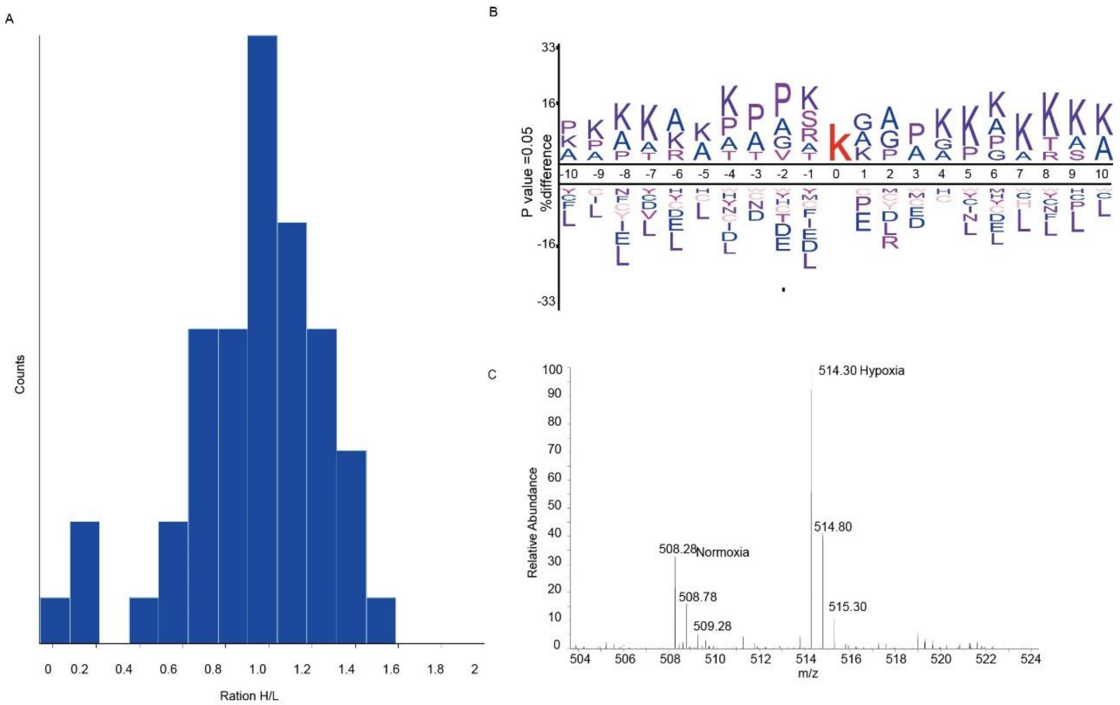

Figure S2. The landscape of histone hypoxia lactylation triggered by hypoxia in ESCC cells, related to Figure 2.

(A) Histogram showing peptides in KYSE30 cells cultured in hypoxia and normoxia.

(B) Sequence motif logo shows a representative sequence for Kla sites.

(C) The MS/MS spectra of H3K9la peptides which were marked by $^{13}C_6$-lys and $^{12}C_6$-lys in hypoxia or normoxia.

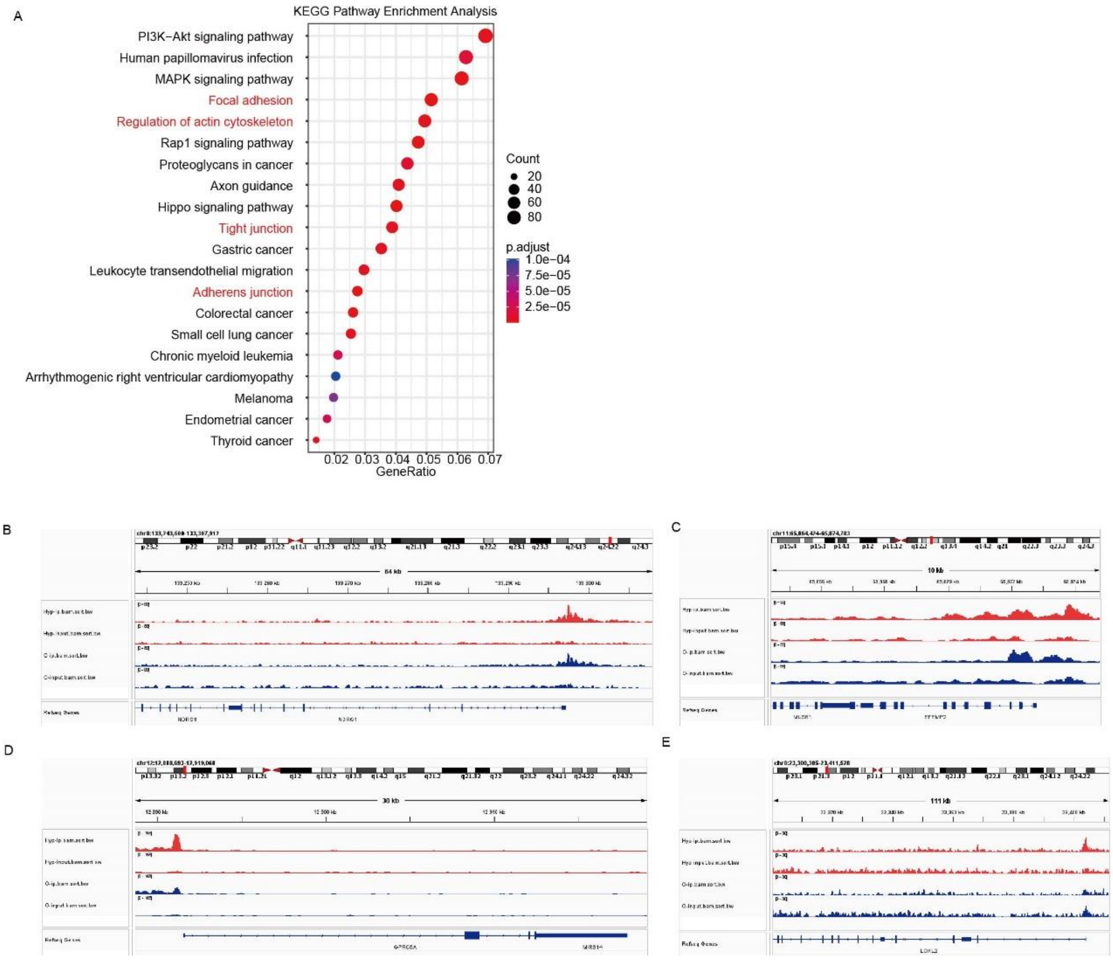

Figure S3. Genome-wide analysis of the transcriptional consequences of H4K9la in KYSE30 cells cultured in normoxia and hypoxia, related to Figure 3.

(A) KEGG enrichment analysis of differentially expressed genes in KYSE30 cells cultured in normoxia and hypoxia.

(B-E) *GPRC5A, LOXL2, EFEMP2* and *ITGA5* promoter in the genomic position was identified to enrich in H3K9la peaks when KYSE30 cells cultured in hypoxia.

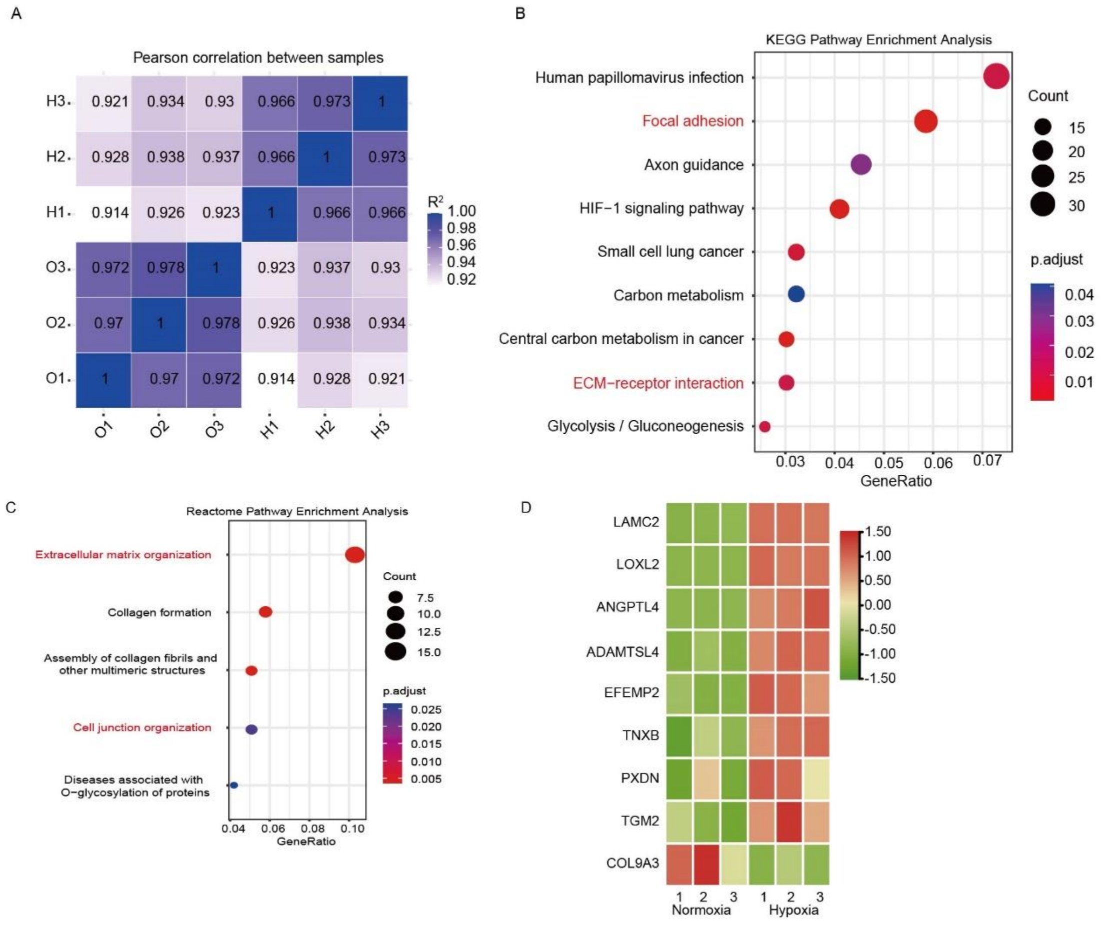

Figure S4. RNA-seq combined with H3K9la MNase ChIP-seq and proteomics analysis in KYSE30 cells cultured in hypoxia, related to Figure 4.

(A) Heatmap showed Pearson correlation between hypoxia (H) group RNA-seq data and normoxia (O) group RNA-seq data.

(B) KEGG enrichment analysis of differentially expressed genes in KYSE30 cells cultured in normoxia and hypoxia.

(C) Reactome enrichment analysis of the overlapped genes.

(D) Heatmap showed the collagen-containing extracellular matrix related genes in RNA-seq which promoters in the genomic position was identified to enrich in H3K9la peaks.

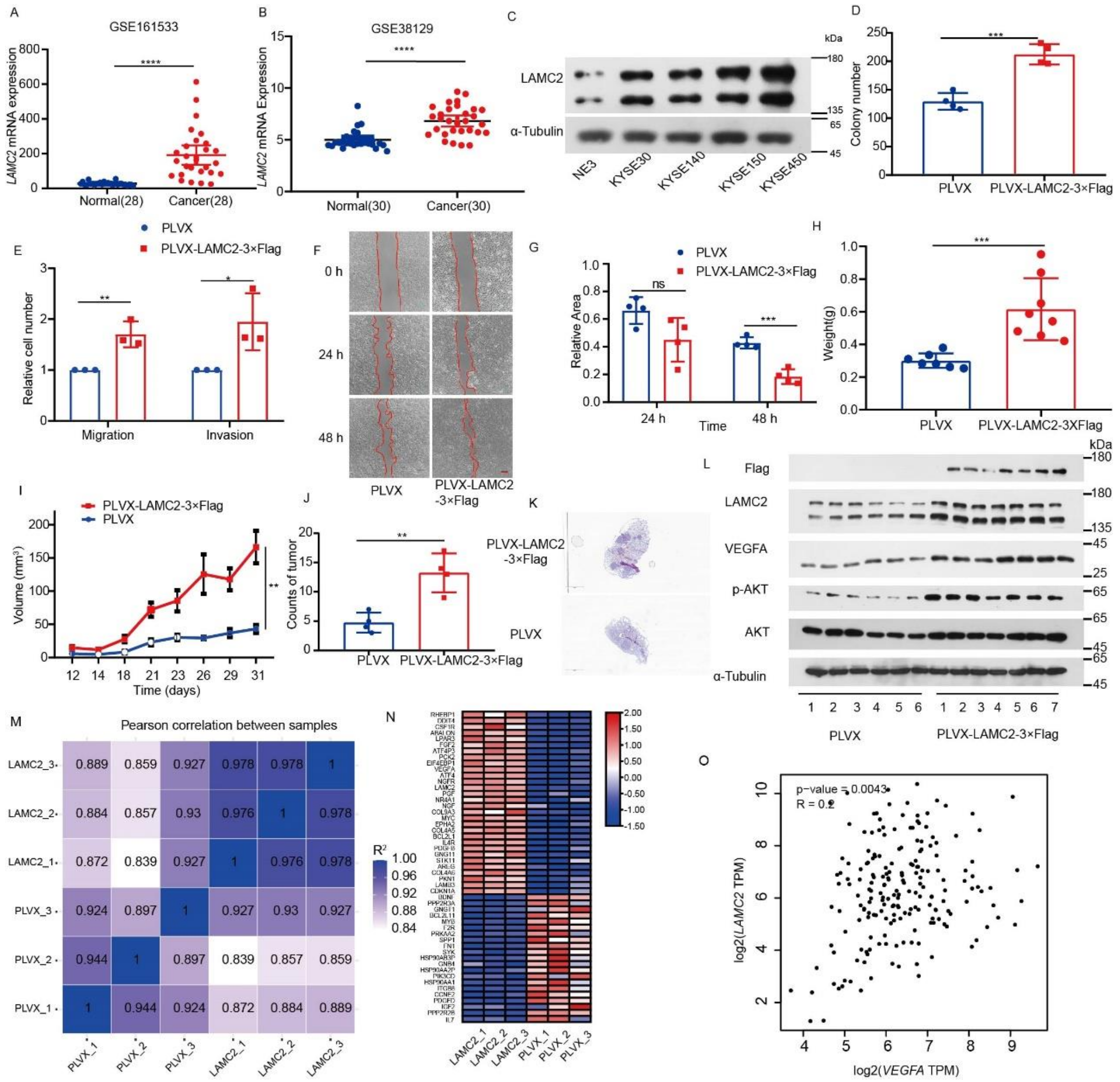

Figure S5. LAMC2 plays an oncogenic role in ESCC.

(A, B) GSEA data showed that LAMC2 mRNA levels was highly expressed in ESCC tumors compared to the normal controls, related to Figure 6.

(C) LAMC2 was highly expressed in these ESCC cell lines compared with NE3 cell line.

(D) Colony formation assay of KYSE30 cells with overexpression of LAMC2 (n = 3 per group).

(E) Transwell assays were used to examine the migratory and invasive abilities of KYSE30 cells with knockdown of LAMC2 (n = 3 per group).

(F,G) Wound healing assay show the motility of KYSE30 cells with overexpression of LAMC2. Bar = 100 μm (n = 5 per group).

(H) The weight of the tumors was counted (PLVX group = 6, PLVX-3×Flag group = 7).

(I) The tumor sizes were continuously recorded to draw tumor growth curves (PLVX group = 6, PLVX-3×Flag group = 7).

(J) Metastatic tumor foci in lung were observed (n = 4 per group).

(K)Hematoxylin-eosin staining of metastatic tumor foci in lung. The scale bar shows 2 mm.

(L) The LAMC2 increased the level of VEGFA and p-AKT in xenograft tumor tissues.

(M) Heatmap showed Pearson correlation between LAMC2 overexpressed group RNA-seq data and control group RNA-seq data.

(N) Heatmap showed the PI3K-Akt signaling pathway related genes in RNA-seq.

(O) TCGA-ESCC data showed that the expression of LAMC2 were related with VEGFA.

Statistical significance was analyzed by *Student's t-test.* Data are mean ± SEM. *$P < 0.05$, **$P < 0.01$., ***$P < 0.001$, ****$P < 0.0001$, ns indicates no significance.

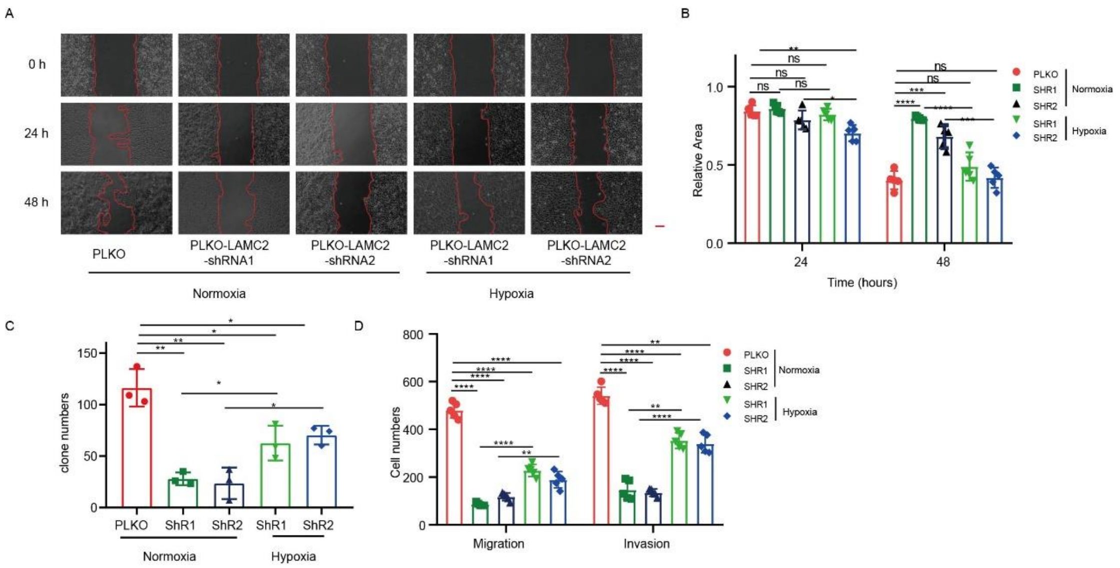

Figure S6 LAMC2 activates the PI3K/Akt signaling pathway to influence the expression of VEGFA, related to Figure 7.

(A, B) Wound healing assay show the motility of KYSE30 cells with knockdown of LAMC2 in hypoxia. Bar = 100 μm (n = 5 per group).

(C) Colony formation assay of KYSE30 cells with knockdown of LAMC2 in hypoxia (n = 3 per group).

(D) Transwell assay show the migratory and invasive abilities of KYSE30 cells with knockdown of LAMC2 in hypoxia. The scale bar shows 100 μm (n = 5 per group). Statistical significance was analyzed by *Student's t-test.* Data are mean ± SEM. *$P < 0.05$, **$P < 0.01$., ***$P < 0.001$, ****$P < 0.0001$, ns indicates no significance.
